# An integrative review of the methodology and findings regarding dietary adherence in end stage kidney disease

**DOI:** 10.1186/s12882-017-0734-z

**Published:** 2017-10-23

**Authors:** Kelly Lambert, Judy Mullan, Kylie Mansfield

**Affiliations:** 1 Department of Clinical Nutrition, Wollongong Hospital, Illawarra Shoalhaven Local Health District, Level 5, Block C, Crown Street, Wollongong, NSW 2500 Australia; 2 Centre for Health Research Illawarra Shoalhaven Population (CHRISP), Australian Health Services Research Institute, University of Wollongong, iC Enterprise 1, Innovation Campus, Wollongong, New South Wales 2522 Australia; 3 School of Medicine, Faculty of Science, Medicine and Health, University of Wollongong, Northfields Ave, Wollongong, New South Wales 2522 Australia

**Keywords:** Dietary adherence, Self-management, End stage kidney disease, Adherence, Compliance, Chronic kidney disease, Dialysis; fluid restriction, Potassium, Phosphate

## Abstract

**Background:**

Dietary modification is an important component of the management of end stage kidney disease (ESKD). The diet for ESKD involves modifying energy and protein intake, and altering sodium, phosphate, potassium and fluid intake. There have been no comprehensive reviews to date on this topic. The aims of this integrative review were to (i) describe the methods used to measure dietary adherence (ii) determine the rate of dietary adherence and (iii) describe factors associated with dietary adherence in ESKD.

**Methods:**

The Web of Science and Scopus databases were searched using the search terms ‘adherence’ and ‘end stage kidney disease’. Of the 787 potentially eligible papers retrieved, 60 papers of 24,743 patients were included in this review. Of these papers, 44 reported the rate of dietary adherence and 44 papers described factors associated with adherence.

**Results:**

Most of the evidence regarding dietary adherence is derived from studies of hemodialysis patients (72% of patients). The most common method of measuring dietary adherence in ESKD was subjective techniques (e.g. food diaries or adherence questionnaires). This was followed by indirect methods (e.g. serum potassium, phosphate or interdialytic weight gain). The weighted mean adherence rate to ESKD dietary recommendations was 31.5% and 68.5% for fluid recommendations. Adherence to protein, sodium, phosphate, and potassium recommendations were highly variable due to differences in measurement methods used, and were often derived from a limited evidence base. Socioeconomic status, age, social support and self-efficacy were associated with dietary adherence. However, factors such as taste, the impact of the diet on social eating occasions; and dietetic staffing also appear to play a role in dietary adherence.

**Conclusion:**

Dietary adherence rates in people with ESKD are suboptimal. Further research is required on dietary adherence in patients with ESKD from different social, educational, economic and ethnic groups. This research may identify other factors which may impact upon adherence, and could be used to inform the design of future strategies to improve dietary adherence. Future research that reports not just the rate of adherence to individual components of the nutrient prescription but also the overall quality of the diet would be useful.

## Background

The prevalence of Chronic Kidney Disease (CKD) is increasing rapidly [1]. Driven by an aging population and increasing rates of obesity, diabetes and hypertension, approximately 1 in 8 adults globally are known to have CKD [2]; and it is estimated that about 2% of these individuals with CKD will progress to End Stage Kidney Disease (ESKD) [3]. An appropriate diet can slow progression of CKD to ESKD [4]; ameliorate the complications of CKD and ESKD [5–8], and increase survival [9, 10], making dietary modification a critical part of the management of CKD and ESKD [11].

There is no standard renal diet. Instead, a progressive accumulation of dietary restrictions occurs as patients’ progress from CKD to ESKD. Typically, people with early CKD need to modify their intake of protein and sodium. In contrast, people with ESKD need to modify their intake of kilojoules; their fluid and protein intake; reduce their intake of minerals, such as sodium, potassium and phosphate; and potentially increase their intake of vitamins and minerals, such as vitamin C, B, folate, B12 and zinc [12]. Because of the large number of dietary modifications required, the diet for people with ESKD is considered by dietitians to be one of the most complex and restrictive therapeutic diets [13, 14]. Adults with ESKD also perceive diet to be complicated and contradictory to typical healthy eating advice [15, 16]. For example, fruits, vegetables and dairy products are often restricted in ESKD due to their potassium or phosphate content.

In addition to these challenges, the diets for people with CKD and ESKD (hereafter referred to as the renal diet for simplicity) also changes when patients commence or change the type of renal replacement therapy. For example, people receiving hemodialysis are routinely required to restrict dietary potassium intake, whereas those undertaking peritoneal dialysis are not (27). These subtle differences in the renal diet prescription, combined with conflicting dietary advice between health professionals [16], are often cited as an ongoing source of frustration, bewilderment and confusion for people with ESKD [16, 17]. Given the challenges imposed by the renal diet, it is unsurprising that dietary adherence is often reported to be poor [18, 19].

Adherence, also used interchangeably with the term ‘compliance’, is frequently cited as: “the degrees to which patient behaviours coincide with the recommendations of health care providers” ([20], page S188). Previous researchers have investigated adherence to various ESKD treatment components, such as medications [21]; phosphate binders [22]; hemodialysis attendance [23], and peritoneal dialysis treatments [24]. However, dietary adherence in people with ESKD is more complex and has not been explored in detail. The limited evidence that is available suggests that dietary adherence rates vary greatly between studies [25]. It is also unclear if adherence varies between the individual nutrients modified in the dietary regimen for people with ESKD. A better understanding of dietary adherence in ESKD is critical because poor dietary adherence is associated with worse health outcomes [26, 27]. Improved knowledge and understanding of the issues associated with renal diet adherence may translate to improved dietary management strategies and improved health outcomes. Therefore, the aim of this integrative review is to provide a comprehensive summary of the evidence regarding dietary adherence in people with ESKD. The specific research questions posed in this integrative review were:

1. What methods have been used to measure dietary adherence in adults with ESKD?
2. What is the estimated rate of dietary adherence in adults with ESKD?
3. What factors are associated with dietary adherence in adults with ESKD?

## Methods

Integrative reviews provide a comprehensive understanding of a complex phenomenon by synthesising qualitative and quantitative literature [28]. To increase rigour, this integrative review utilised methodology described by previous authors [29, 30]. In brief, this methodology includes clearly delineating the focus of the research question/s, undertaking a well-defined literature search strategy, systematically evaluating studies and compiling a transparent collation of findings.

### Literature search

Comprehensive searches of the Web of Science and Scopus databases were conducted during April 2015. The key words ‘adherence’ and ‘end stage kidney disease’ were used to identify suitable peer reviewed journal articles. The corresponding MeSH terms and Boolean operators used to retrieve articles in these searches are shown in Table 1. The reference lists of retrieved studies and review articles were also hand searched for additional relevant publications.

**Table 1:** Search terms used in integrative review of dietary adherence in end stage kidney disease

Search term	MeSH terms used
Adherence	adheren*OR non adheren* OR non-adheren* OR complian* OR non complian*
End stage kidney disease	end stage kidney failure OR end stage renal failure OR end stage renal disease

* indicates truncation to find variations of root term

### Inclusion criteria

Studies considered eligible for inclusion were any experimental, observational or qualitative studies that included (i) human adults with ESKD (stage 4 or 5 CKD, conservatively managed or on any renal replacement therapy modality); (ii) reported either the rate of dietary adherence or examined factors associated with dietary adherence; (iii) reported the results in English and (iv) were available in full text. Editorials, practice guidelines, review articles, paediatric studies, studies not in English and studies not reporting the rate of dietary adherence were excluded from the analyses. Dates of publication were restricted to 2000–2015. This coincided with the release date of the first clinical practice guidelines for the nutritional management of chronic kidney disease [31].

### Data extraction

Extracted data from the eligible included studies were compiled into three summary tables to assist with interpretation and synthesis of the results. Table 2 is comprised of all studies included in this integrative review and contains a description of the salient features of each study. Table 3 contains the rates of adherence to the renal diet. Table 4 outlines the factors associated with dietary adherence in ESKD.

**Table 2:** Summary table of studies describing rates or factors associated with dietary adherence in ESKD (*n* = 60 studies of 24,743 patients)

Authors	Patient numbers	Location	ESKD group	Type of study	Approach used to measure adherence	Methods used to measure adherence	Reports adherence rate	Reports factors associated with adherence
Agondi et al., 2011 [51]	117	Brazil	HD	Cross sectional study	Combination	IDWG, FFQ		✓
Ahrari et al., 2014 [38]	237	Iran	HD	Cross sectional study	Subjective	DDFQ	✓	✓
Antunes et al., 2010 [47]	79	Brazil	HD & PD	Prospective observational study	Subjective	3 day food record	✓	
Baraz et al., 2010 [59]	63	Iran	HD	RCT	Indirect	Blood tests	✓	✓
Barnett et al., 2007 [62]	26	Malaysia	HD	Pre post intervention	Indirect	IDWG	✓	
Casey et al., 2002 [63]	21	England	HD	Prospective observational study	Indirect	IDWG	✓	
Chan et al., 2012 [88]	188	Malaysia	HD	Cross sectional study	Combination	DDFQ, bloods, IDWG	✓	✓
Chan et al., 2010 [39]	173	Hong Kong	PD	Cluster analysis	Subjective	DDFQ	✓	✓
Chen et al., 2006 [48]	70	China	PD	Prospective cohort study	Subjective	3 day food record		✓
Clark-Cutaia et al., 2014 [44]	122	USA	HD	Secondary analysis of baseline RCT data	Combination	IDWG, 3 day food recall		✓
DeBrito-Ashurst et al., 2011 [34]	20	England	CKD	Qualitative study using focus groups	Subjective	Focus group		✓
DeBrito-Ashurst et al., 2013 [61]	56	England	CKD	RCT	Indirect	Urine specimen		✓
Dowell et al. 2006 [32]	4	USA	HD	Pre post intervention	Subjective	Food diary		✓
Durose et al., 2004 [72]	71	UK	HD	Cross sectional study	Indirect	Blood tests	✓	✓
Elliot et al., 2015 [84]	95	USA	HD	Cross sectional study	Combination	PAPM, blood tests	✓	✓
Ford et al. 2004 [73]	70	USA	HD	Pre post intervention	Indirect	Blood tests		✓
Gordon et al., 2010 [36]	88	USA	KT	Qualitative interviews	Subjective	Self-report	✓	✓
Gordon et al., 2009 [35]	82	USA	KT	Qualitative interviews	Subjective	Self-report	✓	✓
Harvinder et al., 2013 [45]	245	Malaysia	HD & PD	Cross sectional study	Subjective	2 day food recall	✓	
Hecking et al., 2004 [78]	3039	Europe^a^	HD	Prospective observational study	Indirect	Blood tests, IDWG	✓	
Hollingdale et al., 2008 [13]	20	England	NDCKD & dialysis	Qualitative study using two focus groups	Subjective	Focus group		✓
Johansson et al., 2013 [49]	106	England	HD & PD	Cross sectional study	Subjective	3 day food record	✓	✓
Kara et al., 2007 [40]	160	Turkey	HD	Cross sectional study	Subjective	DDFQ	✓	✓
Karavetian et al., 2014 [91]	570	Lebanon	HD	RCT	Subjective	3 day food recall, DNAQ		✓
Khalil et al., 2011 [76]	100	USA	HD	Cross sectional study	Combination	DDFQ, bloods, IDWG	✓	✓
Khalil & Darawad, 2014 [87]	190	Jordan	HD	Cross sectional study	Combination	DDFQ, bloods, IDWG	✓	
Khoueiry et al., 2001 [52]	70	USA	HD	Cross sectional study	Subjective	FFQ	✓	
Kugler et al., 2011 [41]	456	Germany & USA	HD	Cross sectional study	Subjective	DDFQ	✓	✓
Kugler et al., 2005 [33]	916	Germany & Belgium	HD	Cross sectional study	Subjective	DDFQ	✓	✓
Lam et al., 2010 [42]	173	Hong Kong	PD	Cross sectional study	Subjective	DDFQ	✓	✓
Lee et al., 2002 [56]	62	Hong Kong	HD	Cross sectional study	Combination	Self-report, bloods, IDWG	✓	✓
Lindberg et al., 2009 [64]	4498	Sweden	HD	Retrospective observational study	Indirect	IDWG	✓	✓
Mellon et al., 2013 [19]	50	Ireland	HD	Cross sectional study	Indirect	Blood tests, IDWG	✓	✓
Molaison et al. 2003 [65]	316	USA	HD	RCT	Indirect	IDWG	✓	✓
Mason et al., 2014 [60]	47	Australia	NDCKD	Cross sectional study	Indirect	Urine specimen	✓	
Mok et al. 2001 [55]	50	Hong Kong	HD	Cross sectional study	Subjective	Stress scale		✓
Moreira et al., 2013 [77]	130	Portugal	HD	Prospective observational study	Subjective	3 day food record	✓	
Morales Lopez et al., 2007 [58]	34	USA	HD	Cross sectional study	Indirect	Blood tests, IDWG	✓	✓
O’Connor et al., 2008 [66]	73	Scotland	HD	Prospective observational study	Indirect	IDWG	✓	✓
Paes-Barreto et al., 2013 [43]	89	Brazil	NDCKD	RCT	Subjective	24 h food recall	✓	✓
Pang et al., 2001 [67]	92	China	HD	Cross sectional study	Indirect	IDWG	✓	✓
Park et al., 2008 [80]	160	South Korea	HD	Cross sectional study	Indirect	Blood tests, IDWG	✓	✓
Poduval et al., 2003 [74]	117	USA	HD	Cross sectional study	Indirect	Blood tests		✓
Quan et al., 2006 [50]	30	China	PD	Prospective observational study	Subjective	3 day food record	✓	✓
Russell et al., 2011 [57]	19	USA	HD	Pre post intervention	Indirect	Blood tests, IDWG	✓	
Rocco et al., 2002 [46]	1000	USA	HD	Analysis of baseline results of RCT	Combination	2 day food recall, bloods	✓	
Sagawa et al., 2001 [93]	10	Japan	HD	Pre post intervention	Combination	IDWG, 5 day food record		✓
Saran et al., 2003 [27]	7676	USA, Europe, Japan	HD	Prospective observational study	Indirect	Blood tests, IDWG	✓	✓
Sharp et al. 2005 [68]	56	Scotland	HD	RCT	Indirect	IDWG	✓	✓
Sutton et al., 2001 [82]	34	England	PD	Cross sectional study	Subjective	5 day food record	✓	
Thomas et al. 2001 [92]	276	USA	HD	Cross sectional study	Subjective	Diet screen questionnaire		✓
Tsay et al., 2003 [69]	62	Taiwan	HD	RCT	Indirect	IDWG		✓
Unruh et al., 2005 [75]	739	USA	HD	Prospective observational study	Indirect	Blood tests	✓	
Vlaminck et al., 2001 [37]	564	Belgium	HD	Cross sectional study	Subjective	DDFQ	✓	
Wang et al., 2003 [53]	266	Hong Kong	PD	Cross sectional study	Subjective	7 day FFQ	✓	✓
Wang et al., 2007 [54]	249	Hong Kong	PD	Cross sectional study	Subjective	7 day FFQ	✓	
Welch et al. 2001 [70]	148	USA	HD	Cross sectional study	Indirect	IDWG	✓	✓
Yokoyama et al. 2009 [71]	72	Japan	HD	Cross sectional study	Indirect	IDWG		✓
Yusop et al., 2013 [81]	90	Malaysia	HD	Cross sectional study	Subjective	2 day food recall	✓	
Zrinyi et al. 2003 [102]	107	Hungary	HD	Cross sectional study	Subjective	RABQ		✓

Legend: *CKD* Chronic Kidney Disease any stage, *DDFQ* Dialysis Diet and Fluid Non Adherence Questionnaire [36], *DNAQ* Dietary Non Adherence Questionnaire [90], *ESKD* End Stage Kidney Disease, *FFQ* food frequency questionnaire, *HD* Hemodialysis, *IDWG* Interdialytic weight gain, *KT* Kidney transplant, *ND-CKD* Non dialysing end stage chronic kidney disease, *PAPM* Precaution Adoption Process Model [83], *PD* Peritoneal dialysis, *RCT* Randomised Control Trial, *RABQ* Renal Adherence Behaviour Questionnaire [105]

^a^France, Germany, Italy, Spain, UK

**Table 3:** Rates of dietary adherence in ESKD (*n* = 44 studies of 23,177 patients)

				Reported dietary adherence rate (%)									
Authors, Year, Country	N / gender % male	CKD stage / RRT modality	Adherence Measurement Tool	Renal diet	Fluid	Energy	Protein	PO4	K	Na	Fat	CHO	Fibre
Ahrari et al., 2014, Iran [38]	237 / 57.7	HD	DDFQ	58.9	54.8								
Antunes et al., 2010, Brazil [47]	79 / 60.7	HD & PD	3 day food recall				43.0						
Baraz et al., 2010, Iran [59]	63 / 52.4	HD	Serum urea, uric acid creatinine, K, PO4	64.0									
Barnett et al., 2007, Malaysia [62]	26 / 50.0	HD	IDWG		47.0								
Casey et al., 2002, England [63]	21 / 52.0	HD	IDWG		61.9								
Chan et al., 2012, Hong Kong [88]	188 / 48.9	HD	DDFQ	36.2	48.4								
			Serum K, PO4	27.7									
			IDWG		24.5								
Chan et al., 2010, Hong Kong [39]	76 / 39.5	PD	DDFQ	65.8	85.0								
	77 / 68.8			44.2	66.2								
Durose et al. 2004, United Kingdom [72]	71 / 58.0	HD	Serum PO4, K and IDWG		77.0			69.0	96.0				
Elliott et al., 2015, USA [84]	95 / 57.0	HD	PAPM					32.6					
			Serum phosphate					43.8					
Gordon et al., 2009, USA [35]	82 / 57.3	KT	Self-report		33.0								
Gordon et al., 2010, USA [36]	88 / 58.0	KT	Self-report		35.0								
Harvinder et al., 2013, Malaysia [45]	52 / 51.0^a^	PD	2 day food recall			11.0	21.0						
	38	PD				23.0							
	107 / 59.0^b^	HD				25.0	33.0						
	48	HD				16.0							
Hecking et al., 2004, UK [78]	620 / 62.0	HD	Serum phosphate, potassium and IDWG		96.6			77.1	90.2				
Hecking et al., 2004, Spain [78]	576 / 57.0				92.5			77.4	72.7				
Hecking et al., 2004, Italy [78]	600 / 57.0				82.3			84.5	72.0				
Hecking et al., 2004, France [78]	571 / 84.6				94.4			61.5	84.6				
Hecking et al., 2004, Germany [78]	672 / 57.0				85.7			78.7	89.1				
Johannson et al., 2013, England [49]	106 / 71.7	HD & PD	3 day food record			20.0	60.0						
Kara et al., 2007, Turkey [40]	160 / 57.5	HD	DDFQ	49.1	31.9								
Khalil et al., 2011, USA [76]	100 / 44.0	HD	DDFQ	66.0	50.0								
			Serum bloods	44.0			99.0	48.0	90.0				
			IDWG		9.0								
Khalil and Darawad, 2014, Jordan [87]	190 / 54.0	HD	DDFQ	27.0	23.0								
			Serum bloods	46.0			20.0	83.0	80.0				
			IDWG		50.0								
Khoueiry et al., 2001, USA [52]	70 / 54.0	HD	FFQ				31.4			48.6	T:7.1 SF:31.4	94.3	2.9
Kugler et al., 2011, Germany and USA [41]	456 / 57.9	HD	DDFQ	19.6	25.7								
Kugler et al., 2005, Germany and Belgium [33]	916 / 52.9	HD	DDFQ	18.6	25.4								
Lam et al., 2010, Hong Kong [42]	173 / 51.0	PD	DDFQ	38.0	64.0								
Lee et al., 2002, Hong Kong [56]	62 / 50.0	HD	Self-report	66.0	63.0								
			Serum PO4, K	35.0				43.5	61.0				
			IDWG		40.3								
Lindberg et al., 2009, Sweden [64]	4498 / 60.3	HD	IDWG		70.0								
Mellon et al., 2013, Ireland [19]	50 / 60.0	HD	Serum PO4, K and IDWG		38.0			72.0	66.0				
Molaison et al., 2003, USA [65]	316 / 50.6	HD	IDWG		24.6								
Mason et al., 2014, Australia [60]	47 / 51.1	NDCKD	Urine							32.0			
Moreira et al., 2013, Portugal [77]	130 / 63.8	HD	3 day food record			25.4	67.7						
Morales Lopez et al., 2007, USA [58]	17 / 35	HD	Serum albumin, PO4, K and IDWG				76.0	88.0	65.0				
	17 / 35						59.0	88.0	76.0				
O’Connor et al., 2008, Scotland [66]	73 / 60.3	HD	Serum PO4, IDWG		30.0				84.0				
Paes-Barreto et al., 2013, Brazil [43]	43 / 51.2	HD	24 h food recall				46.5						
	46 / 52.2						37.0						
Pang et al., 2001, China [67]	92 / 42.4	HD	IDWG		68.0								
Park et al., 2008, South Korea [80]	64 / 56.3	HD	Serum PO4, K and IDWG		54.7			68.8	76.6				
	96 / 40.6				37.2			44.8	71.9				
Poduval et al., 2003, USA [74]	117 / 52.1	HD	Calcium Phosphate product					42.0					
Quan et al., 2006, China [50]	30 / 46.7	HD	3 day food record	19.5									
Russell et al., 2001, USA [57]	19 / 47.0	HD	Serum albumin, PO4 and IDWG		78.9		100.0	68.4					
Rocco et al., 2002, USA [46]	1000 / 46.4	HD	2 day food recall			24.0	39.0						
			enPCR				48.0						
Saran et al., 2006, USA [27]	3359 / 55.1	HD	Serum PO4, K, and IDWG		83.2			84.6	93.7				
Saran et al., 2006, Europe [27]	2337 / 59.7				89.0			87.2	80.0				
Saran et al., 2006, Japan [27]	1980 / 62.4				65.5			87.9	92.4				
Sharp et al., 2005, Scotland [68]	56 / 67.9	HD	IDWG		0.0								
Sutton et al., 2001, England [82]	34 / 70.6	PD	5 day food record			11.8	21	70.6					
Unruh et al., 2005, USA [75]	739 / 53.7	HD	Serum PO4, K					59.1	79.3				
Vlaminck et al., 2001, Belgium [37]	564 / 49.1	HD	DDFQ	18.0	28.0								
Wang et al., 2003, Hong Kong 53]	266 / 52.3	PD	7 day FFQ			25.5	39.1						
Wang et al., 2007, Hong Kong [54]	249 / 50.6	PD	7 day FFQ				75.0				T:51.0 SF:84.0	80.0	
Welch et al., 2001, USA [70]	148 / 52.0	HD	IDWG		33.8								
Yusop et al., 2013, Malaysia [81]	90 / 48.9	HD	2 day food recall		31.1	20.0	24.4	82.2	100.0	86.7			
*Total number participants*	*23,177*	*Weighted mean adherence rate*		*31.5*	*68.5*	*23.1*	*45.5*	*79.8*	*85.6*	*61.4*	*TF:41.4* *SF:72.5*	*83.1*	*2.9*

Legend: ^a^gender for total PD group; ^b^gender proportion for total HD group; *CKD* Chronic Kidney Disease, *CHO* adherence to recommendations for carbohydrate intake, *DDFQ* Dialysis Diet and Fluid Non Adherence Questionnaire, *enPCR* equilibrated normalized protein catabolic rate, *FFQ* food frequency questionnaire, *HD* hemodialysis, *IDWG* interdialytic weight gain, *K* adherence to low potassium diet, *KT* kidney transplant; Na: adherence to recommendations for sodium intake: *NDCKD* non-dialysing adults with ESKD; *PAPM* Precaution Adoption Process Model tool, *PO4* adherence to low phosphate diet, *PD* peritoneal dialysis, *Renal diet* refers to adherence to all components of the renal diet prescription, *RRT* renal replacement therapy type; T: adherence to recommendations for total fat intake; SF: adherence to recommendations for saturated fat intake; serum bloods: combination of serum potassium, phosphate and / or others (eg albumin or urea)

**Table 4:** Summary of weighted mean adherence rates for components of the dietary prescription for ESKD

ESKD dietary adherence component	Weighted mean adherence rate (%)	Evidence base
Adherence to fluid recommendations	68.5	28 studies of 20,244 adults with ESKD
Adherence to energy intake recommendations	23.1	7 studies of 1871 adults with ESKD
Adherence to protein intake recommendations	45.5	15 studies of 3701 adults with ESKD
Adherence to the low phosphate diet	79.8	15 studies of 12,571 adults with ESKD
Adherence to the low potassium diet	85.6	12 studies of 12,284 adults with ESKD
Adherence to the reduced sodium diet	61.4	3 studies of 207 adults with ESKD
Adherence to total fat intake recommendations	41.4	2 studies of 319 adults with ESKD
Adherence to saturated fat intake recommendations	72.5	2 studies of 319 adults with ESKD
Adherence to carbohydrate intake recommendations	83.1	2 studies of 319 adults with ESKD
Adherence to fibre recommendations	2.9	1 study of 70 adults with ESKD
Adherence to the renal diet	31.5	13 studies of 3832 adults with ESKD

## Results

**Fig. 1:**
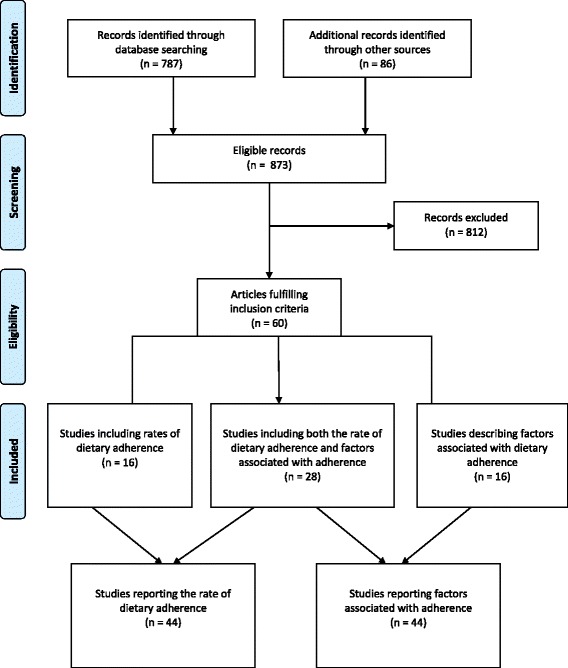
Flowchart illustrating selection of articles for review

The number of potential articles relevant for review was 787 (see Fig. 1). An additional 85 articles were identified after hand searching the references. Following the removal of duplicates and irrelevant articles, a total of 60 articles were included in this review. Of the 60 studies, 16 reported the rate of dietary adherence; 28 studies reported both the rate of adherence and factors associated with adherence; and 16 studies only contained details regarding factors associated with adherence (Fig. 1). For the final synthesis of findings, a total 44 articles reported the rate of dietary adherence, and 44 articles described factors associated with dietary adherence in ESKD.

A summary of the 60 studies included in this integrative review are shown in Table 2. Overall, a total of 24,743 adults with ESKD were studied, and sample sizes in the studies varied from 4 people [32] to more than 7000 [27]. Most of these studies were conducted in Asia (17 studies, 28%) or the USA (16 studies, 27%), followed by studies conducted in the United Kingdom (9 studies, 15%) and Europe (8 studies, 13%) (Table 2). Two studies were transcontinental in nature involving the USA and Germany [33]; as well as Europe, the USA and Japan [27]. The majority of the data on dietary adherence was from studies involving people with ESKD undertaking hemodialysis (43 studies, 72%); followed by people undertaking peritoneal dialysis (7 studies, 12%). Only two studies included people with a kidney transplant (3%). More than half of all included studies were cross-sectional observational studies (*n* = 31 studies, 52%), and only four studies (6%) were qualitative in nature [13, 34–36].

### Methods used to measure dietary adherence in ESKD

Of the 60 articles in this review, a range of approaches to measure dietary adherence were evident. These are summarised in Table 2, and can be broadly categorised into the use of subjective approaches (28 studies, 47%), indirect approaches (23 studies, 38%), and combination approaches (9 studies, 15%).

#### Subjective approaches

Of the 28 studies that used a subjective approach to measuring dietary adherence in ESKD, there were 15 variations of how this was conducted. These are shown in Table 2. The most common method described was the use of the Dialysis Diet and Fluid Non Adherence Questionnaire (DDFQ) [37], a four item self-report instrument that probes the severity and duration of renal diet and fluid restriction non-adherence. This instrument has been demonstrated to be weakly correlated indirect measures of dietary adherence including interdialytic weight gain, serum albumin, serum potassium and serum phosphate [37]. The DDFQ was used as the only method to measure adherence in seven studies [33, 37–42].Other common methods for collecting subjective information about dietary adherence included various iterations of food records such as 24 h recalls [43], 3 day food recalls [44], 2 day food recalls [45, 46], 3 day food records [47–50], and food frequency questionnaires [51–54]. Other subjective methods included the use of stress scales relating to the diet [55] or self-reported adherence [35, 36, 56].

#### Indirect approaches

There were 23 studies that used an indirect approach to measuring dietary adherence. Interdialytic weight gain (IDWG), which refers to the fluid gain in kilograms gained between hemodialysis sessions, was the most frequently reported indirect method for measuring dietary adherence (16 studies, Table 2). This was followed by 10 studies using blood tests to measure serum potassium, phosphate, albumin [57, 58],or urea [59] and urine collections to measure volume or sodium (2 studies, [60, 61]). Ten studies used IDWG in isolation to measure adherence [62–71]. Five studies used only blood tests to measure adherence [59, 72–75].

#### Combination approaches

A combination approach was used in nine studies, with the combination of blood tests, the DDFQ, and IDWG being the most common (Table 2). This type of combination approach theoretically provides information regarding adherence to the overall renal diet, fluid intake and adherence to the low potassium and low phosphate components of the renal diet. Another common combination approach reported was the use of IDWG and food recalls or food records (3 studies).

### Estimated rates of dietary adherence in ESKD

Details regarding the estimated rates of dietary adherence in ESKD were obtained from 44 studies (*n* = 23,117 adults with ESKD). The rates of adherence from the 44 individual studies are shown in Table 3, and the weighted mean adherence rates for the various components of the dietary prescription for ESKD are summarised in Table 4. The weighted mean adherence rates ranged from 2.9% for fibre recommendations to 85.6% for adherence to the low potassium diet (Table 4). The overall rate of adherence to the renal diet was estimated to be 31.5%.

Attempts to compare dietary adherence rates within or between the various components of the renal diet are difficult. This is due to the highly heterogeneous nature of the study participants and the varying methods used to determine adherence. For example, as shown in Table 3, the gender balance of males in the studies varied from 35% [58] to 71.7% [49]. Studies also included cohorts with a known history of non-adherence [68], high rates of depression [76], high rates of malnutrition [77] or large numbers of highly illiterate adults with ESKD [39, 56]. Furthermore, studies varied according to whether participants were from a single centre, or were from large multicentre, and/or transcontinental studies. However, to provide some clarity regarding the estimated rates of dietary adherence, the four most frequently reported types of dietary adherence studies are discussed further in the following sections.

#### Fluid restricted diets

Fluid restrictions are recommended for people with ESKD, and are used to prevent fluid overload and pulmonary oedema. Fluid restricted diets are typically in the range of 1000-1500 ml of fluid per day. For those who have received a kidney transplant, fluid restrictions are not recommended and instead a higher fluid intake is suggested (usually >3000 ml per day [35, 36]). Most studies that report adherence to fluid recommendations in this review were conducted using people undertaking hemodialysis (24 studies), and IDWG was the most frequently used method of measuring adherence.

Overall, adherence rates to fluid recommendations varied from as low as 0% in a population known to be non-adherent [68] to as high as 96.6% [78]. The only two studies which examined adherence to fluid recommendations in people undertaking peritoneal dialysis [39, 42], using the DDFQ to measure adherence found that the adherence rates were between 64 and 85%. In contrast, only one third of adults with a kidney transplant self-reported that they were adherent to fluid recommendations [35, 36].

#### Low phosphate diets

Restriction of dietary phosphate intake is recommended for all adults with ESKD in an attempt to lower the deranged serum phosphate levels [79]. Of the 15 studies that reported low phosphate diet adherence rates, the majority (13 studies) used serum phosphate to measure dietary adherence, and found that rates varied between 43.5%–84.5%. More than half of these studies reported an adherence rate of greater than 70%, with younger people having lower adherence rates (44.8%) when compared to older people (68.8%) [80].

Two studies which measured low phosphate diet adherence used food recalls [81] or food records [82] to obtain data on dietary phosphate intake and neither study reported the proportion of inorganic to organic phosphate intake, an important emerging component of dietary phosphate management [83]. In the only study retrieved that compared the rate of adherence to the low phosphate diet using two different methods, Elliott et al. [84], found that adherence was 32.6% when using a self-report survey on adoption of the low phosphate diet (the Precaution Adoption Process Model tool), compared with an adherence rate of 43.8% using serum phosphate.

#### Low potassium diets

A low potassium diet is recommended for adults with ESKD [85], and is used to prevent the potentially fatal complication of chronic hyperkalemia [86]. Serum potassium was the most frequently reported method for measuring adherence to the low potassium diet, and only one study used a food recall to determine low potassium dietary adherence [81]. All 12 studies of low potassium diet adherence were conducted on in people undertaking hemodialysis, highlighting an obvious lack of research regarding low potassium diet adherence in those undertaking home hemodialysis and in those with CKD.

#### Overall renal diet adherence

One challenge of summarising the literature on renal diet adherence is the varying definitions used by previous researchers about what ‘renal diet’ adherence entails. For example, Baraz et al. [59], defined adherence to the renal diet as serum creatinine, sodium, potassium, calcium, phosphate, albumin, urea and uric acid within acceptable limits. In contrast, Quan et al. [50], defined renal diet adherence as ‘following the dietitian’s prescription’. Despite these differences, the reported adherence rates to the renal diet were relatively poor overall, with a weighted mean adherence rate of 31.5%. Only five of the eighteen cohorts studied achieved an adherence rate greater than 50% ([38, 39, 56, 59, 76]. The measurement tools used to determine renal diet adherence also varied, with five different methods used to describe renal diet adherence: serum measures [59], the DDFQ [33, 37–42], the 3 day food record [50], or a combination of measures including self-report [56, 76, 87, 88]. Furthermore, four studies compared overall renal diet adherence using two different methods: the DDFQ and serum measures [76, 87, 88] or self-report and serum measures [56]. The findings indicated that renal diet adherence varied in the same cohort of adults with ESKD by 8.9% [88] to 31% [56], suggesting that simply using different adherence measurement methods can also affect the adherence rate results.

### Factors reported to be associated with dietary adherence in adults with ESKD

Adherence to medical treatment is a complex process influenced by many social, individual, cultural and environmental factors (83). This component of the integrative review utilised data from 44 studies. To assist with interpretation of the results, the factors reported to be associated with dietary adherence have been categorised according to the WHO Multidimensional Adherence Model [89], and are shown in Table 5. The categories outlined in the WHO model [89] are (i) socioeconomic factors (ii) condition related factors (iii) therapy related factors (iv) health care team and system factors and (v) patient related factors.

**Table 5:** Factors associated with dietary adherence in adults with ESKD categorised according to WHO criteria [88]

Authors	Patient numbers	ESKD group	Socioeconomic factors	Condition related factors	Therapy related factors	Health care team and system related factors	Patient related factors
Agondi et al., 2011 [51]	117	HD	Higher education level Older age		Shorter dialysis vintage Dietary knowledge		Positive beliefs regarding the benefits of the diet
Ahrari et al., 2014 [38]	237	HD					Social and family support
Baraz et al., 2010 [59]	63	HD	Higher education level Being employed Younger age				
Chan et al., 2012 [88]	188	HD	Retired or not working Female gender Older age		Dietary knowledge Short dialysis vintage Diet complexity		Self-efficacy
Chan et al., 2010 [39]	173	PD				Nurse support for home dialysis patients	
Chen et al., 2006 [48]	70	PD			Recipe modification knowledge		
Clark-Cutaia et al., 2014 [44]	122	HD	Male gender Older age				
DeBrito-Ashurst et al., 2011 [34]	20	CKD					Taste preferences & palatability Strategies to manage the diet at social events Positive beliefs & attitudes about the diet
DeBrito-Ashurst et al., 2013 [61]	56	CKD			Recipe modification knowledge		
Dowell et al. 2006 [32]	4	HD			Self-monitoring		
Durose et al., 2004 [72]	71	HD		Knowledge of medical complications of dietary non-adherence	Dietary knowledge		
Elliot et al., 2015 [84]	95	HD	Minimum of high school education White ethnicity	Better quality of life	Shorter dialysis vintage		Perceived benefits of dietary adherence Self-efficacy
Ford et al. 2004 [73]	70	HD				Intensive patient education	
Gordon et al., 2009 [35]	82	KT	Adequate family income		Self-monitoring Dietary knowledge		Taste preferences & palatability Strategies to manage the diet at social events Positive beliefs & attitudes about the diet
Gordon et al., 2010 [36]	88	KT	Male gender Private health insurance Being married	Better self-rated health			High self-efficacy Positive beliefs & attitudes about the diet
Hollingdale et al., 2008 [13]	20	NDCKD & dialysis			Consistent dietary advice / dietary messages		Strategies to manage the diet at social events Positive beliefs & attitudes about the diet
Johansson et al., 2013 [49]	106	HD & PD	Higher socioeconomic status	Better quality of life			Absence of depression Presence of social support
Kara et al., 2007 [40]	160	HD	Older age Being married				Presence of family support Presence of social support
Karavetian et al., 2014 [91]	570	HD			Dietary knowledge	Adequate dietitian staffing Experienced renal dietitian	
Khalil et al., 2011 [76]	100	HD					Absence of depression
Kugler et al., 2011 [41]	456	HD	Lower education level Female gender Being married				Non-smoking status
Kugler et al., 2005 [33]	916	HD	Female Gender Older Age		Short dialysis vintage		Family support Non-smoker Non-diabetic status
Lam et al., 2010 [42]	173	PD	Retired occupational status Low education level Female gender Older age		Dialysis vintage >3 years		
Lee et al., 2002 [56]	62	HD	Unemployment or non-working status		Shorter dialysis hours per week		Positive attitudes to diet High residual renal function >300 ml day
Lindberg et al., 2009 [64]	4498	HD	Older age		Short dialysis vintage		Higher BMI
Mellon et al., 2013 [19]	50	HD	Older age				Perception that diet fits into lifestyle Strategies to manage the diet at social events Positive beliefs & attitudes about the diet
Molaison et al. 2003 [65]	316	HD	Older age Female gender		Self-monitoring		
Mok et al. 2001 [55]	50	HD			Long dialysis vintage		
Morales Lopez et al., 2007 [58]	34	HD	Adequate finances		Culturally appropriate format of patient education Dietary knowledge	Presence of a dietitian on staff	Presence of family support
O’Connor et al., 2008 [66]	73	HD	Female gender Older age				Adequate psychological coping ability
Paes-Barreto et al., 2013 [43]	89	NDCKD			Dietary knowledge	Intensive patient education	
Pang et al., 2001 [67]	92	HD	Lower family income				Lower comorbid disease burden Presence of social support
Park et al., 2008 [80]	160	HD	Older age				Malnutrition
Poduval et al., 2003 [74]	117	HD	College education		Education about food composition		
Quan et al., 2006 [50]	30	PD				Nurse support for home dialysis patients Intensive patient education	
Sagawa et al., 2001 [93]	10	HD			Self-monitoring		
Saran et al., 2003 [27]	7676	HD	Unemployed Male gender Older age Married		Long dialysis vintage	Presence of a dietitian on staff	Family support Non-smoking status
Sharp et al. 2005 [68]	56	HD				Intensive patient education	Higher self-efficacy
Thomas et al. 2001 [92]	276	HD	White ethnicity Female gender		Dietary knowledge practical shopping skills		Family support Positive beliefs & attitudes about the impact of the diet
Tsay et al., 2003 [69]	62	HD			Self-monitoring		High self-efficacy
Wang et al., 2003 [53]	266	PD					No history of fluid overload
Welch et al. 2001 [70]	148	HD					Positive beliefs & attitudes about the impact of the diet
Yokoyama et al. 2009 [71]	72	HD				Dialysis staff encouragement	Lower perceived burden of the diet High self-efficacy Good mental health
Zrinyi et al. 2003 [102]	107	HD	Female gender				High self-efficacy

#### Socioeconomic factors

Twenty four studies provided information on socioeconomic factors associated with dietary adherence. From these studies, age, gender and education level were the most frequently explored socioeconomic factors (Table 5). Older adults and individuals with a higher level of education were consistently associated with greater dietary adherence. Evidence regarding occupation level suggests that those who are not working are more likely to adhere to the renal diet. In contrast, results regarding the relationship between gender and dietary adherence were mixed. Overall, female gender was associated with greater dietary adherence to the renal diet in eight of eleven studies. One of the few studies which reported the opposite result, that is, males were more likely to be adherent to the renal diet, came from the largest study cohort included in this integrative review with more than 7000 adults with ESKD [27].

#### Condition and therapy related factors

Information on condition and therapy related factors associated with dietary adherence were obtained from 25 studies (Table 5). From these studies, most evidence supported an association between the length of time undertaking hemodialysis and poorer renal diet adherence [27, 64, 88]. Reasons for this remain unexplored, but it is thought to be related to the practical challenge of managing the complex dietary modifications required for many years [64], and to the scale of modifications required to long standing behaviours [90].

The relationship between dietary knowledge and renal diet adherence is not clear and the evidence base comes from only 6 studies of less than 2000 adults with ESKD [35, 43, 72, 88, 91, 92]. Poor dietary knowledge was associated with suboptimal renal diet adherence in four studies [35, 88, 91, 92]. Provision of renal diet related practical skills and knowledge, such as learning food composition details [74], self-monitoring strategies [32, 35, 69, 93] or learning appropriate recipe modifications [48, 61] were found to be associated with greater renal diet adherence and were also highly valued by patients in the three qualitative studies [13, 34, 35]. Factors such as receiving conflicting dietary advice from different health professionals [13], and the complexity of the diet [88] were reported to be associated with poorer dietary adherence.

#### Health care team and system factors

Research on the relationship between the health care team and health care system factors on dietary adherence in ESKD is scarce, but of increasing academic interest [89, 94]. Evidence from nine studies suggests that the quality of the relationship between the patient and the health care professional is important (Table 5). For example, patients with EKSD who receive intensive education from experienced renal dietitians [73, 91], or patients who received support from renal health professionals [39, 50, 71] were more adherent to the renal diet. Furthermore, inadequate support or infrequent contact from renal dietitians was specifically found to impact negatively on dietary adherence [27, 58, 91]. The main reason suggested by the authors for these findings was inadequate staffing ratios [27, 91]. This is an important finding as staffing surveys of renal dietitians from the US [95, 96], UK [97], Asia [98] and Australia [99, 100] consistently report that renal dietitian staffing ratios are below evidence based practice recommendations.

#### Patient related factors.

Evidence for patient related factors was obtained from 25 studies with ESKD. Factors such as the presence of social and family support, and positive beliefs and attitudes towards the renal diet were frequently studied and found to be consistently associated with improved renal diet adherence. Patients who understood and valued the potential benefits of dietary modification [19, 34–36, 70, 92] were more adherent to the diet than those who felt the diet posed a burden [71]. Self-efficacy refers to a person’s confidence to control their behaviour to achieve a goal [101].The impact of self-efficacy on dietary adherence was investigated in six studies, and these studies reported that adults exhibiting greater self-efficacy also experienced higher dietary adherence rates [68, 69, 71, 84, 88, 102].

The impact of the renal diet on social eating events was also a specific patient related factor identified with renal diet adherence in four studies [13, 19, 34, 35]. Findings from the three qualitative studies [13, 34, 35] indicated several situational or contextual factors relating to social eating that impacted on dietary adherence. For example, dietary adherence was influenced by acceptance of the renal diet by family members or friends [13, 34]. One study also reported that patients were not adherent to the diet to avoid ridicule from others or because foods adherent to the renal diet were not readily available when eating out [35].

Taste preferences (particularly for salt) were also reported as a barrier to renal diet adherence in several studies [34, 35, 88]. For example, De Brito-Ashurst et al. [34] reported perceptions that salt was a vital food ingredient and thus not possible to reduce in the diet without reducing palatability [34]. Finally, depression appears to be an under researched area pertaining to renal diet adherence. This is surprising given the high prevalence of the disorder in patients with ESKD [103]. Two studies explored the relationship between depression and renal diet adherence [49, 76], those who were depressed also exhibited worse dietary adherence. Similarly, those with greater mental health [71] or adequate psychological coping skills [66] were more likely to adhere to the renal diet.

## Discussion

Adherence to medical treatment is considered to be the most effective method for improving health outcomes [104]. The intent of this integrative review was to synthesise the body of evidence regarding dietary adherence in adults with ESKD and identify the factors which influence dietary adherence. This review has yielded four key findings that can be used by clinicians and researchers to improve renal diet adherence.

The first key finding of this review was that research on dietary adherence in ESKD is dominated by studies using subjective self-reported information. Measurement of dietary adherence in ESKD is challenging, and unlike medication or dialysis related adherence studies, there is no ‘gold standard’ or single physiological marker exists that indicates a person is consuming the recommended ESKD diet prescription. Subjective methods such as diet recalls, food frequency questionnaires and diet records impose a significant subject burden in an unwell population. They are also known to be associated with problems of underreporting of dietary intake [105]. Adherence questionnaires like the DDFQ [37] or the Renal Adherence Behaviour questionnaire [106] also assume patients have adequate cognitive capabilities and appropriate levels health literacy; as well as an adequate understanding of the diet to answer the questions appropriately. This is particularly problematic given that cognitive impairment and low health literacy are common in patients with ESKD [107–111]. Consequently, subjective approaches should also be used with caution in those with ESKD.

The second key finding of this review is that indirect physiological measures (such as serum potassium, phosphate or interdialytic weight gain) have been used frequently to measure dietary adherence in ESKD. The obvious advantages of using serum markers are that they are relatively cheap, easy to obtain, and have a low patient burden. However, serum potassium and phosphate are strongly influenced by non-dietary factors such as residual renal function [112, 113], constipation [114]; adherence to prescribed medications [115, 116], acid base balance [117] and time between treatments [118], making them unreliable and inaccurate markers of dietary adherence [119–121]. Future studies of dietary adherence in ESKD should ideally attempt to use direct observation and immediate quantification of dietary intake to provide the most accurate data on dietary intake. However, limited staffing, finances, and the inability to monitor patients for long time periods, make this approach unlikely to be implemented. For pragmatic reasons it is therefore suggested that a combination of indirect measures (eg interdialytic weight gain, urine volume and sodium) and subjective methods (such as dietitian assisted dietary recalls [122]) be used instead to increase the rigour of the information collected [89, 123]. Improved reporting of dietary outcomes in future studies is also needed and future research should include comprehensive details of dietary intake as well as reporting the rate of adherence. This approach has been used in several recent studies [124, 125], and provides superior quality information that could then be used to guide future dietary adherence interventions.

This review provides clinicians with estimates of the rate of adherence to the renal diet and is the third important finding of this review. Attempts to compare the estimated dietary adherence rates to other components of the ESKD treatment regimen are challenging however, because the renal diet contains many components. Overall, the weighted mean adherence rates to fluid, phosphate, potassium and carbohydrate recommendations were similar to rates of adherence in other medical conditions. For example, it is estimated that 50–70% of patients are expected to be adherent to their therapy irrespective of the disease, prognosis or setting [123, 126, 127]. Previous research in people with chronic diseases (such as diabetes, hypertension or ischemic heart disease) [128, 129]; or on other ESKD self-management components [120, 130, 131] have also reported adherence rates of this magnitude. However, the low rate of adherence to the overall renal diet as well as to specific components such as energy, protein, sodium, total fat and fibre reported in this review suggests that designing interventions to improve dietary adherence in those with ESKD is required [132]. Interventions to improve adherence are proposed to have a greater impact on patient health than any further improvements in medical technologies and treatments [89].

The final important findings of this review were that there are several factors that are associated with good dietary adherence: older age; higher education levels; the presence of social or family support; and high levels of self-efficacy. Several other unique factors such as taste, the impact of the diet on social eating occasions; and dietetic staffing also play a role in dietary adherence.

However, several factors impacting on dietary adherence in ESKD examined in this review warrant specific further discussion. For example, the relationship between renal diet knowledge and renal diet adherence requires further investigation. Previous studies of adherence in people with ESKD have demonstrated that knowledge was strongly associated with adherence to the ESKD treatment regimen [23, 133, 134]. However in the present review, greater knowledge of the renal diet was not always associated with improved dietary adherence [72]. This surprising finding is consistent with a recent systematic review on the relationship between dietary knowledge and dietary adherence in general, which also showed that in adults there was only a weak association [135]. In other words, it appears that knowledge alone is not sufficient for optimal renal dietary adherence [65, 136]. Several emerging areas that may explain these findings include the possibility that individuals with ESKD may have lower levels of patient activation [137] and patient engagement [138] for undertaking the changes required when following the renal diet, and therefore further investigation of the reasons for these findings is clearly warranted.

The quality of the relationship between the patient and the health care provider was identified in this review as an important modifier of dietary adherence. In addition, recent evidence indicates that multidisciplinary care slows the rate of decline in renal function [139], suggesting that adherence rates may be better in patients treated by multidisciplinary teams. Further research exploring how this relationship impacts on dietary adherence is important and could be used to redesign dietary education strategies. Patients with kidney disease have expressed dissatisfaction with the information provided to them by health care providers in numerous studies [16, 140–143]. As a result, patients now use the internet to seek answers to the questions they feel are important to them [140, 142–145]. Whether this occurs with those seeking renal diet information remains unexplored, and the impact of “googling” on dietary adherence is unknown. Similarly, frustrations have been expressed by patients about receiving contradictory dietary information [13, 16], but how this impacts on dietary adherence is also unknown. The perceptions by patients and other staff about the role of the renal dietitian should also be explored further. For example, patients are commonly referred to renal dietitians by medical staff to prevent disease progression or to control side effects [146–148]. However, these are infrequently expressed motivators for attending dietitian appointments or for adhering to the diet [17]. Instead, patients report consulting renal dietitians to either improve their quality of life, or to decrease the negative impact of the diet on social eating occasions [17, 149].

The impact of factors such as health literacy and cognitive impairment on dietary adherence in ESKD also requires further exploration. The renal diet is acknowledged as one of the most complex diets to teach, understand and implement [14]. The presence of cognitive impairment and low health literacy in patients with ESKD could contribute to the poor rates of dietary adherence reported in this review. Previous research has confirmed that health literacy skills and cognitive capabilities are important influences on other self-management abilities in patients with ESKD [150–154]. It seems reasonable therefore, to assume that a poor understanding of the renal diet, poor quality patient education materials or poorly given instructions relating to the diet may lead to errors in the dietary self-management process and worsen health outcomes [150, 152]. Therefore, a better understanding of how these factors impact on dietary adherence is critical for preventing disease progression and further complications.

There are several areas for future research that are evident from this integrative review. For instance, due to the lack of studies on dietary adherence in patients with ESKD not undertaking dialysis, it is recommended that future research on dietary adherence should include this group of patients, as well as kidney transplant recipients. Future studies should also utilise a comprehensive dietitian assisted dietary assessment method such as a diet recall, diet record, FFQ or diet quality index. Exploring differences in adherence that may occur between non-dialysis and dialysis days; as well as the differences in adherence that may occur according to dialysis vintage, or in minority cultural groups are also important. Studies should also investigate differences in adherence to the renal diet according to gender and over time. This is an important area for future research because adherence to the renal diet requires continuous self-regulation and adherence would be expected to vary day to day, as well as over time, between renal replacement therapy modalities and according to season [123, 155]. Future research on renal diet adherence should also consider reporting the impact of the renal diet on overall diet quality [14, 156–158]. The relationship between nutrient modification and overall diet quality is increasingly recognised as important, and is known to influence the risk and development of chronic diseases such as kidney disease [159, 160]. The use of indirect measures will not adequately capture these variations in quality, quantity and adherence [161]. Further research examining how patients make sense of the renal diet, and how this may impact on adherence would also be useful and could be used to inform and guide practioners about the content of future dietary education strategies and patient education resources.

Several recommendations for clinicians are also evident from this review. Additional support or alternative education and counselling strategies may be required to enhance dietary adherence in individuals who are male; younger; with lower education levels, and with inadequate social and family support. Patients that may be depressed have low self-efficacy and those with a long dialysis vintage may also be another target group for additional support from health professionals. Based on the findings of this review, advice from health professionals within renal units where possible should also be consistent, and delivered utilising appropriate health literacy techniques [162, 163]. Clinicians should also consider utilising or expanding upon the use of pragmatic and flexible dietary prescriptions (such as those described recently for individuals requiring a low protein diets [164–166] in an attempt to improve dietary adherence.

The strengths of this review include the exhaustive coverage of the topic using studies retrieved from a comprehensive search of two large databases and the retrieval of a large number of additional relevant articles from reference lists. There are also limitations relating to this review which need to be acknowledged. The grey literature was not searched and articles in languages other than English were not included. The search strategy used was based on MeSH terms, and alternative or additional search terms may have retrieved other relevant articles.

## Conclusions

Dietary modification is an important component of the management of ESKD. Based on the findings of this review it is estimated that around one in three adults with ESKD are adherent to the renal diet and approximately two thirds of adults with ESKD adhere to recommendations regarding fluid. Uncertainty surrounds these results though due to wide variations in adherence rates between studies, and the use of methodological approaches with inherent flaws in reliability and accuracy. Adults found to be most likely to adhere to the renal diet includes females, older adults, and individuals with adequate family and social support and self-efficacy. This review has also highlighted that further research on dietary adherence is required in several cohorts with ESKD, such as kidney transplant recipients or those with ESKD not undertaking dialysis. Developing strategies to address the barriers identified in this review to dietary adherence in ESKD may improve health outcomes.
